# The Effects of Vitamin D on Gentamicin-Induced Acute Kidney Injury in Experimental Rat Model

**DOI:** 10.1155/2013/313528

**Published:** 2013-06-15

**Authors:** Ender Hur, Alev Garip, Asuman Camyar, Sibel Ilgun, Melih Ozisik, Sena Tuna, Murat Olukman, Zehra Narli Ozdemir, Eser Yildirim Sozmen, Sait Sen, Fehmi Akcicek, Soner Duman

**Affiliations:** ^1^Department of Nephrology, Bulent Ecevit University Medical School, Esenkoy, 67100 Zonguldak, Turkey; ^2^Department of Internal Medicine, Ege University Medical School, 35100 Izmir, Turkey; ^3^Department of Pharmacology, Ege University Medical School, 35100 Izmir, Turkey; ^4^Department of Biochemistry, Ege University Medical School, 35100 Izmir, Turkey; ^5^Department of Pathology, Ege University Medical School, 35100 Izmir, Turkey

## Abstract

*Introduction*. Acute kidney injury (AKI) pathogenesis is complex. Findings of gentamicin nephrotoxicity are seen in 30% of the AKI patients. Vitamin D has proven to be effective on renin expression, inflammatory response, oxidative stress, apoptosis, and atherosclerosis. We aimed to investigate the effect of vitamin D in an experimental rat model of gentamicin-induced AKI. *Methods*. Thirty nonuremic Wistar albino rats were divided into 3 groups: Control group, 1 mL saline intramuscular (im) daily; Genta group, gentamicin 100 mg/kg/day (im); Genta + vitamin D, gentamicin 100 mg/kg/day (im) in addition to 1**α**, 25 (OH)_2_D_3_ 0.4 mcg/kg/day subcutaneously for 8 days. Blood pressures and 24-hour urine were measured. Blood urea and creatinine levels and urine tubular injury markers were measured. Renal histology was semiquantitatively assessed. *Results*. Urea, creatinine and urine neutrophil gelatinase-associated lipocalin, and kidney injury molecule-1 were all increased in Genta group indicating AKI model. Systolic blood pressure decreased, but urine volume and glutathione increased in Genta + Vit D group compared to Control group. Histological scores indicating tubular injury increased in Genta and Genta + Vit D groups. *Conclusions*. Vitamin D does not seem to be effective on histological findings although it has some beneficial effects via RAS system and a promising effect on antioxidant system.

## 1. Introduction

Acute kidney injury (AKI) pathogenesis is complex, and promoting events may be completely different (ischemia or toxins are major factors that precipitate in the injury), but similar pathways may be involved in subsequent injury responses. For this, reason to study AKI models, various methods were defined for each specific situation. 

Gentamicin derived from gram-positive bacteria called *Micromonospora* purpurea present in soil and water having potential in treating aerobic gram-negative bacteria. Accumulation of gentamicin in proximal renal tubules may cause nephrotoxicity which leads to brush border network damage [1]. The nephrotoxicity involves renal free radical production and accumulation, consumption of antioxidant defense mechanisms, glomerular congestion, and acute tubular necrosis [2–5], leading to diminished creatinine clearance and renal dysfunction. The pathological mechanisms also involve elevation of endothelin-1, upregulation of transforming growth factor-beta (TGF-*β*), significant increase in monocyte/macrophage infiltration into the renal cortex and medulla, augmentation of oxidative stress, and apoptosis and also necrosis [6–9]. 

Vitamin D is a pleiotropic hormone that affects classical and nonclassical tissues. Its primary sites of action are still considered to be the intestine, bone, and kidneys [10]. A number of studies have shown positive therapeutic efficacy of vitamin D and analogs to reduce proteinuria [11–13]. Recently, a large randomized placebo-controlled clinical trial (the VITAL Study, *n* = 281) confirmed that paricalcitol was able to reduce albuminuria and blood pressure in patients with diabetic nephropathy who were already on renin-angiotensin system inhibitor therapy [14]. Together, these clinical data provide a strong case to argue for the use of vitamin D analogs as a complementary therapy for treatment of proteinuria. Given the importance of podocytes in the regulation of glomerular filtration, it is speculated that podocytes are important antiproteinuric targets of vitamin D [15] although tubular effect of vitamin D is still debate.

Inflammation and reactive oxygen substances play an important role on acute kidney injury pathophysiology. Vitamin D has already known antiinflammatory and immunomodulatory effects. In the present study, the aim was to investigate whether vitamin D might be a useful therapeutic agent for gentamicin-induced AKI model in rats. Up to now vitamin D related protection mechanisms on AKI remain to be fully proven. Given the complexity of the disease and the pleiotropic nature of the agent activity, the protective effect would be expected and be of a multifactorial nature. 

## 2. Methods

### 2.1. Study Protocol

Thirty nonuremic Wistar albino male rats (*n* = 30; weight 180–220 g) were divided into three equal groups. They were housed in polycarbonate cages under 24°C room temperature with a 12-hour light/dark cycle and fed a standard laboratory diet. The Animal Ethics Committee of Ege University Hospital approved the study design. The three groups of rats consisted of the following: Control group, 1 mL saline intramuscular (im) daily; Genta group, gentamicin 100 mg/kg/day (im); Genta + vitamin D, gentamicin 100 mg/kg/day (im) in addition to 1*α*, 25 (OH)_2_D_3_ 0.4 mcg/kg/day subcutaneously for 8 days.

Systolic blood pressure was measured in conscious rats by the indirect tail cuff method, using an electrosphygmomanometer and pneumatic pulse transducer (MAY NIBP200-A, Ankara, Turkey), and 24-hour urine was collected in metabolic cages. After 1 hour, ketamine HCL anesthesia (60 mL/kg body weight) was applied, and immediately, blood samples were collected through direct cardiac puncture in sacrificed rats. Semiquantitative assessment of kidneys was carried out by the same pathologist who was unaware of the groups. 

### 2.2. Functional Parameters

Serum urea and creatinine levels and urine gamma glutamine transferase (GGT) activity were determined using commercial available kits spectrophotometrically (Biolabo Reagents, Maizy France). Urine (Lipocalin-2)-Neutrophil gelatinase-associated lipocalin (NGAL) (Boster Biological Technology Co, Ltd.), and kidney injury molecule-1 (KIM-1) (Adipo Bioscience, Inco, USA) levels were measured with ELISA kits. Urine glutathione (GSH) levels were determined by a commercially available kit (Cayman Chemical Company, Ann Arbor, MI, USA).

### 2.3. Structural Parameters

Four percent formalin was used for fixation of kidney samples which were embedded into paraffin wax. Paraffin blocks were divided into sections 5 *μ*m in thickness, and hematoxylin-eosin and Masson's were trichrome used for staining. All samples were examined by the same pathologist who was unaware of which samples originated from which group. Tubular degeneration, necrosis, tubule interstitial nephritis, and total histological scores were evaluated semiquantitatively from 0 to 3. 

Tubular degeneration (TD): in the cytoplasm of the proximal tubule epithelial cells, stained bodies of various sizes and vacuolization containing acidophilus were considered as TD.

 Scoring:
 Absence of TD; 0
 Mild TD: small and a few focus TD in immediately beneath the capsule (0%–10); 1
 Moderate TD: for a few focal focus TD and along the tubular segment (10%–25); 2
 Severe TD: diffuse and significant TD along the tubular segment (% 25–50); 3
 Very severe TD: TD was greater than 50%; 4

Tubular necrosis (TN): defined as loss of epithelial cells of the nucleus, dark acidophilic cytoplasm, loss of tubular epithelial cells into tubular lumen, and acellular sections of tubules.

 Scoring:
 Absence of TN; 0
 Mild TN: small and a few focus TN in immediately beneath the capsule (0%–10); 1
 Moderate TN: for a few focal focus TN and along the tubular segment (10%–25); 2
 Severe TD: diffuse and significant TN along the tubular segment (% 25–50); 3
 Very severe TN: TN was greater than 50%; 4

Tubulointerstitial inflammation (TIN): defined as infiltration of inflammatory cells in perivascular and interstitial areas.

 Scoring:
 Absence of TIN; 0
 Mild TIN: a few pieces of infiltration concentrated on perivascular area (0–5%); 1
 Moderate TIN: usually infiltrations involved in cortical interstitial and many focal areas (5–10%); 2
 Severe TIN: diffuse and significant infiltration areas (15–25%); 3
 Very severe TIN: TIN was greater than 50%; 4

Total histologic score (THS): TD/2 + TN + TIN/2, respectively.

 Normal THS: 0–2
 Mild THS: 2–5
 Moderate THS: 5–8
 Severe TH: >8

### 2.4. Statistical Analysis

Study results are presented as mean ± standard error of the mean (SEM). Nonparametric tests (Kruskal Wallis, Man-Whitney *U* test) were performed as statistical evaluation, and *P* < 0.05 was considered as significant.

## 3. Results 

**Figure 1:**
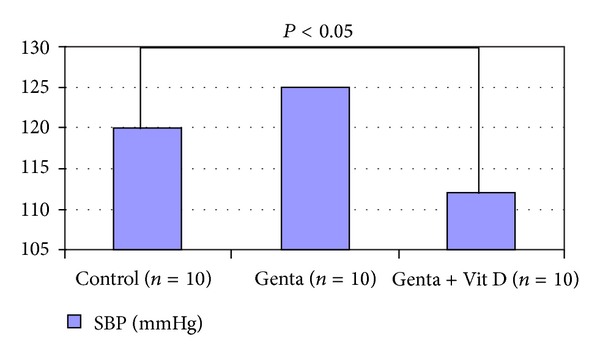
Blood pressures.

**Figure 2:**
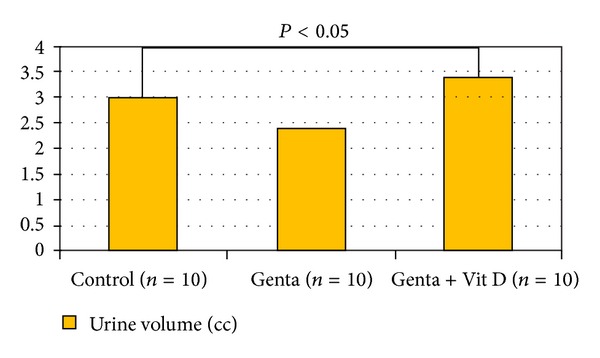
Urine Volumes.

**Figure 3:**
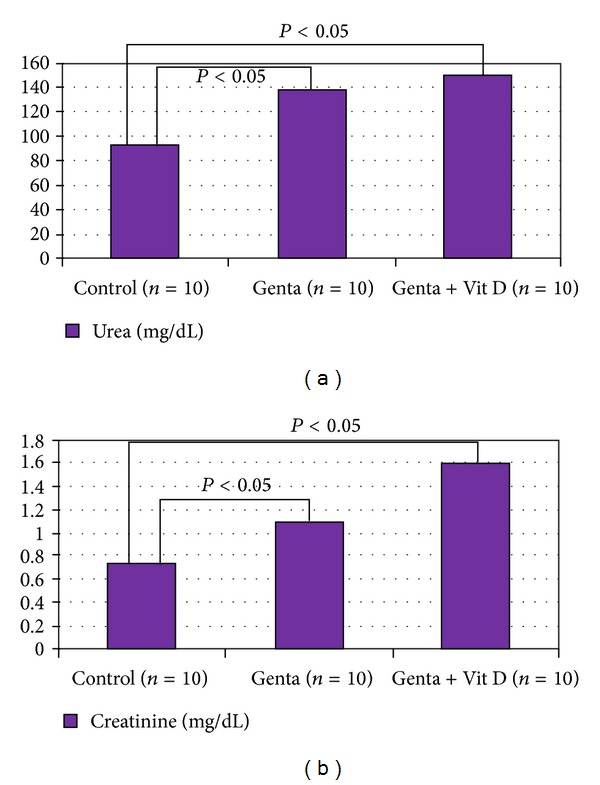
Renal function tests.

**Figure 4:**
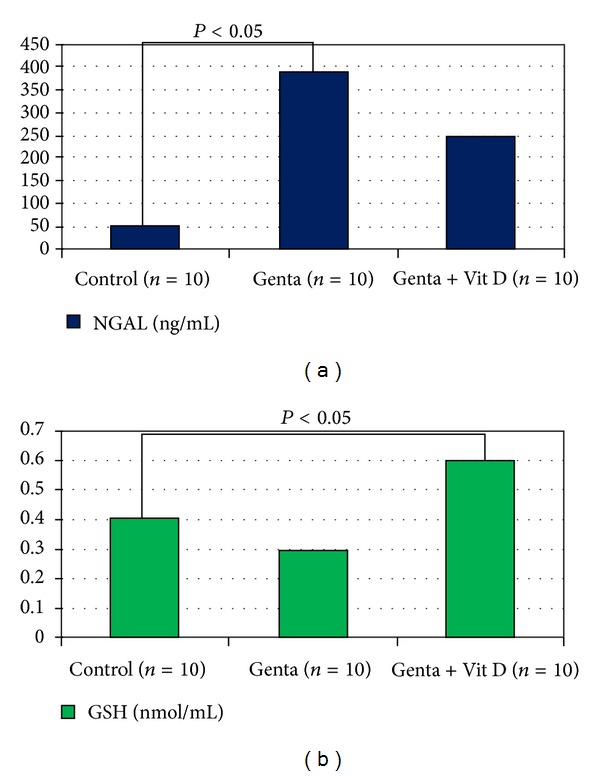
Urine neutrophil gelatinase-associated lipocalin and glutathione levels.

Systolic blood pressure in Control group was 120 ± 6 and decreased to 112 ± 13 mmHg (Figure 1), and urine volume increased in Genta + Vit D group (3.4 ± 0.5 cc) compared to Control group (3 ± 0.5 cc) (*P* < 0.05) (Figure 2). In Control group, urea and creatinine levels were 91 ± 6 and 0.74 ± 0.03 and increased to 137 ± 6 mg/dL and 1.1 ± 0.1 mg/dL in Genta group, also 149 ± 5 mg/dL and 1.6 ± 0.3 mg/dL in Genta + Vit D, indicating acute kidney injury (Figure 3). Neutrophil gelatinase-associated lipocalin (NGAL) was 49.5 ± 7 significantly and increased to 390 ± 143 ng/mL in Genta group and decreased to 247 ± 112 ng/mL in Genta + Vit D group. Glutathione and gamma glutamine transferase were 0.4 ± 0.13 and 1.3 ± 0.35 and increased to 0.6 ± 0.1 nmol/mL and 59 ± 19 U/L in Genta + Vit D group (Figure 4). Kidney injury molecule 1 (KIM-1) level was 0.64 ± 0.05 in Control group and increased significantly in both Genta and Genta + Vit D groups (4.7 ± 0.6 and 6 ± 0.5 ng/mL) (Table 1).

**Figure 5:**
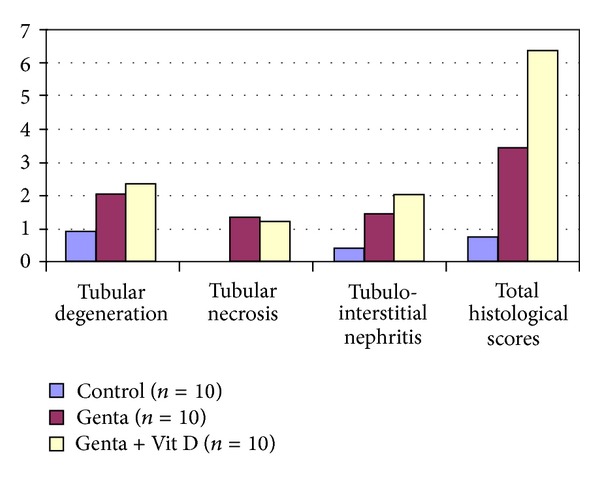
Renal histology. Pathological examination performed by semiquantitatively scored from 0 to 3.

**Figure 6:**
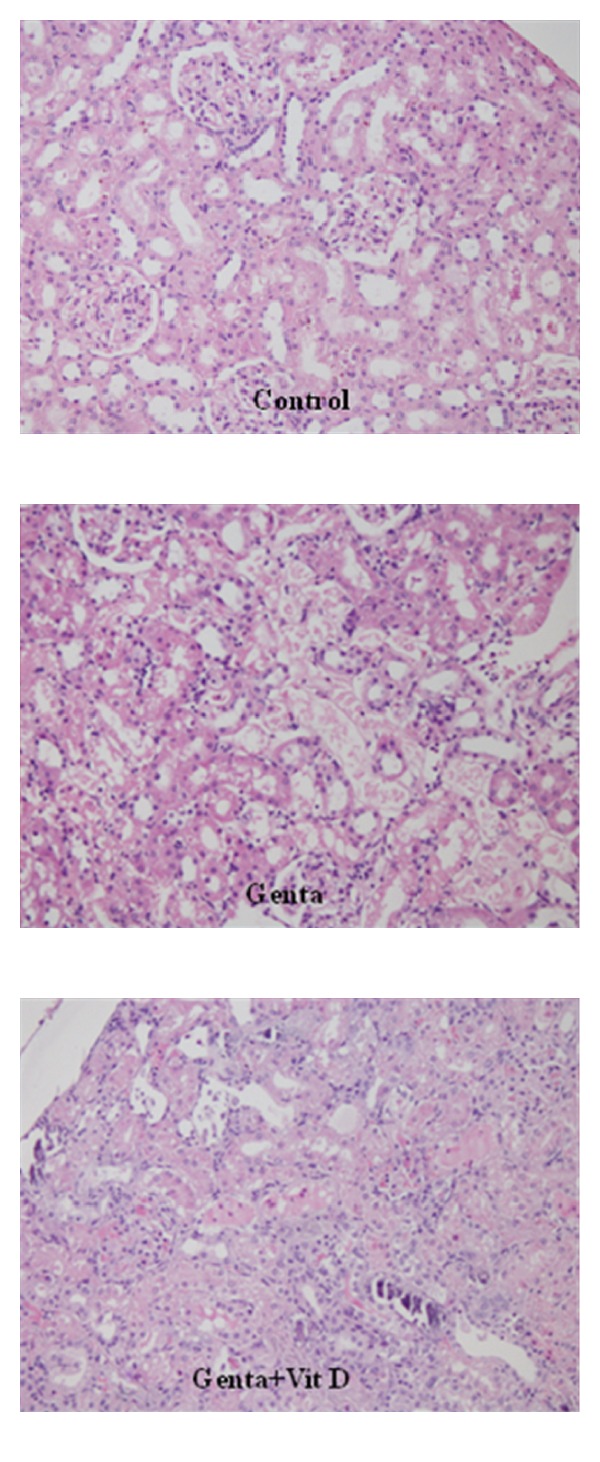
Renal pathology.

Histological scores of tubular degeneration (TD), tubular necrosis (TN), tubulointerstitial nephritis (TIN), and total histological score (THS) all increased significantly in Genta and Genta + Vit D groups compared to Control group (Figures 5 and 6). TIN and THS scores also significantly were higher in Genta + Vit D group compared to Genta group (Table 1).

## 4. Discussion

Gentamicin is a positively charged chemical that strongly binds to the acidic phosphoinositide components of the brush border membrane which is a negatively charged portion of the proximal tubule, and they mainly act on the cationic drug receptor, megalin, located deeply at the base of the brush border villi. The receptor-drug complex thus formed is rapidly internalized by a pinocytosis process and checked up by lysosomes, where lysosomal phospholipidosis occurs that disrupts a number of renal intracellular processes [16, 17].

Renin angiotensin system (RAS) in the kidney is a mandatory mediator of renal injury. Vitamin D hormone has a negative regulatory effect on RAS by suppressing renin expression [18, 19]. It is shown that vitamin D receptor-absent mutant mice develop more severe renal damage (e.g., interstitial fibrosis, increased albuminuria, and glomerulosclerosis) than wild-type counterparts in diabetic state [20] or under postrenal acute kidney injury [21], because of enhanced activation of the RAS in the kidney. In 5/6 nephrectomised rats given paricalcitol treatment attenuated tubulointerstitial and glomerular injury and decreased blood pressure and albuminuria by inhibiting the activation of the locally produced RAS in the remnant kidneys [22]. Doxercalciferol had an effect on modulating fat-induced renal injury by targeting the RAS and lipid metabolism [23]. Other studies proved that combination therapy with one RAS inhibitor (ACE inhibitor or ARB) and one vitamin D analog (paricalcitol or doxercalciferol) leads to additive or synergistic therapeutic effects in blocking renal injury in experimental rat models of type 1 and type 2 diabetes mellitus [24–27]. The renal protection of the combination therapy is the inhibition of the compensatory renin induction usually encountered in the use of both RAS inhibitors and the vitamin D analogues. Renin induction inhibition and accumulation of angiotensin II within the kidney leads to excellent therapeutic results [28]. The combination strategy in these studies explains why vitamin D analogs are still effective in reducing albuminuria in CKD patients who are already receiving RAS inhibitors [29, 30]. In present study, we found that SBP is decreased in Genta + vitamin D group compared to Control group. This indicates that the RAS blocking effect of vitamin D was still strong enough even in the presence of acute kidney injury. Increased urine volume in this group also may be attributed to RAS blocking effect of vitamin D therapy.

Functionally, gentamicin-related nephrotoxicity is characterized by a decrease in glomerular filtration rate and high levels of serum creatinine and blood urea, indicating renal dysfunction [31–37]. In the present study, Genta-induced experimental AKI model is formed and proven by an increase of these renal function tests appropriately. Unfortunately, they were still higher in Genta + vitamin D group than in Control group.

In the literature, there are increasing multifactorial mechanisms suggested as the leading cause of gentamicin nephrotoxicity. Lysosomal apoptosis and phospholipidosis have been suggested to play a pivotal role in gentamicin-induced nephrotoxicity [38–40]. In the past, gentamicin was shown to increase reactive oxygen species (ROS) like superoxide anions, hydroxyl radicals and hydrogen peroxides, and reactive nitrogen species generation in the renal cortex that eventually lead to renal structural and functional deterioration [41–44]. Further, it is linked with marked increases in lipid peroxidation levels [45], nitrotyrosine formation [46] and protein oxidation [47]. In our study, we demonstrated that in Genta group although it did not reach statistical significance a little GSH decrease occurred. On the other hand, Genta + Vit D group had a statistically significant GSH increase. In the literature, gentamicin has been also shown to cause changes in the composition of lipid membranes executed by free radicals mediated lipid peroxidation [48]. Furthermore, gentamicin-administered rat kidneys are more susceptible to ROS damage because of the induction of deficiency in antioxidant defense enzymes like superoxide dismutase and catalase [49, 50]. Here in our study, vitamin D might have some beneficial effect on gentamicin-induced AKI by increasing GSH levels and acting as an antioxidant mechanism, and also NGAL levels were not increased unlike to Genta group. 

Structurally, gentamicin-related nephrotoxicity is associated with the edema of proximal tubular cells, glomerular hypertrophy, perivascular edema, inflammation, glomerular congestion, cellular desquamation, glomerular atrophy, tubular necrosis, and tubular fibrosis [40, 51–57]. Gentamicin causes macrophage infiltration and higher transforming growth factor-*β* which may lead to progression of TIN [40]. In the present study, TIN scores were significantly higher in Genta group, but surprisingly in Genta + Vit D group, histological scores were even higher than Genta group. Tubular histological parameters all were increased in Genta group indicating experimental AKI model occurred but unfortunately all these parameters were not decreased in Genta + Vit D group. 

Acut kidney injury as a result of gentamicin-induced tubular necrosis stimulates inflammatory events by recruiting intercellular adhesion molecule-1 and monocyte chemo-attractant protein-1 at the site of injury that enhance the migration of monocytes and macrophages to the site of tissue damage, ultimately leading to renal pathogenesis [58, 59]. In present study we demonstrated that GGT levels in Genta + Vit D group and KIM-1 levels in both Genta and Genta + Vit D groups were increased indicating that renal tubular damage occurred in Genta groups and also even using Vit D did not prevent progression of injury.

## 5. Conclusion

In the past, vitamin D was shown as an effective drug on podocytes preventing proteinuria, regulate bone remodeling, regulate cell cycles, and the renin-angiotensin system [60]. The present study indicates that the progression of gentamicin-induced AKI was not stopped by vitamin D treatment shown by histological findings although it probably has some beneficial effects on the RAS system via blood pressure lowering and increase of urine volume and a promising effect on antioxidant system. As a result given the various overlapping pathways involved in AKI pathogenesis, intended therapies may need to use vitamin D in addition to other therapeutical approaches to target diverse pathways in order to achieve success. 

## Figures and Tables

**Table 1:** Clinical and laboratory findings.

	Control, *n* = 10	Genta, *n* = 10	Genta + Vit D, *n* = 10
SBP (mmHg)	120 ± 6	125 ± 10	112 ± 13^a^
DBP (mmHg)	68 ± 4	75 ± 7	74 ± 8
Urine volume (cc)	3 ± 0.5	2.4 ± 0.4	3.4 ± 0.5^a^
Urea (mg/dL)	91 ± 6	137 ± 6^a^	149 ± 5^a^
Creatinine (mg/dL)	0.74 ± 0.03	1.1 ± 0.1^a^	1.6 ± 0.3^a^
NGAL (ng/mL)	49.5 ± 7	390 ± 143^a^	247 ± 112
GSH (nmol/mL)	0.4 ± 0.13	0.3 ± 0.04	0.6 ± 0.1^a^
GGT (U/L)	1.3 ± 0.35	38 ± 37	59 ± 19^a^
KIM-1 (ng/mL)	0.64 ± 0.05	4.7 ± 0.6^a^	6 ± 0.5^a^
TD	0.9 ± 0.1	2 ± 0^a^	2.3 ± 0.2^a^
TN	0	1.3 ± 0.2^a^	1.2 ± 0.2^a^
TIN	0.4 ± 0.2	1.4 ± 0.2^a^	2 ± 0.1^ab^
THS	0.75 ± 0.15	3.4 ± 0.4^a^	6.3 ± 0.7^ab^

SBP: systolic blood pressure; DBP: diastolic blood pressure; NGAL: neutrophil gelatinase-associated lipocalin; GSH: glutathione; KIM: kidney injury molecule; TD: tubular degeneration; TN: tubular necrosis; TIN: tubulointerstitial nephritis; THS: total histological score; *P* < 0.05, ^a^Group versus Control, ^b^Group versus Genta.
